# Repeated cognitive assessments show stable function over time in patients with ALS

**DOI:** 10.1007/s00415-024-12479-x

**Published:** 2024-06-09

**Authors:** Linn Öijerstedt, Juliette Foucher, Anikó Lovik, Solmaz Yazdani, Alexander Juto, Ulf Kläppe, Fang Fang, Caroline Ingre

**Affiliations:** 1 Department of Clinical Neuroscience, Karolinska Insitutet, 171 77 Stockholm, Sweden; 2 Department of Neurology, Karolinska University Hospital, Stockholm, Sweden; 3 Institute of Environmental Medicine, Karolinska Institutet, Stockholm, Sweden; 4 Institute of Psychology, Leiden University, Leiden, The Netherlands

**Keywords:** Amyotrophic lateral sclerosis, Cognitive impairment, ECAS, Survival, Longitudinal

## Abstract

**Background:**

Amyotrophic lateral sclerosis (ALS) is a multisystem disorder with not only motor symptoms but also extra-motor features including cognitive impairment. The most common cognitive profile observed in patients with ALS includes deficits in executive function, language, and social cognition. However, longitudinal studies on cognitive changes over time in ALS are sparse. We aimed to investigate the presence and nature of cognitive impairment at the time of ALS diagnosis and its association with survival as well as explore longitudinal cognitive change.

**Method:**

Patients (*n* = 216) were recruited at the Karolinska University Hospital in Stockholm, Sweden. Follow-up visits (*n* = 307 in total) were performed every 6 months. Cognitive impairment was assessed using the Edinburgh Cognitive and Behavioural ALS Screen (ECAS) and/or Montreal Cognitive Assessment (MoCA).

**Results:**

Cognitive impairment was observed in 38% of the patients at the time of ALS diagnosis, and the majority of these patients had deficits in executive function and/or language. Patients with cognitive impairment at the time of diagnosis had a more rapid decline in ALSFRS-R at 12- and 18-months follow-up, and a shorter survival. Cognitive function was stable during the first 2 years after diagnosis, and did not follow the trajectories of decline in motor functions.

**Conclusion:**

Cognitive impairment in ALS was associated with a faster decline of motor functions, and shorter survival. However, cognitive function did not deteriorate over time. Cognitive assessment is essential for the patients and caregivers to understand the phenotypic expression of ALS.

**Supplementary Information:**

The online version contains supplementary material available at 10.1007/s00415-024-12479-x.

## Introduction

Amyotrophic lateral sclerosis (ALS) is a progressive neurodegenerative disease affecting upper and lower motor neurons. ALS is conceptualised as a multisystem disorder including not only motor symptoms but also extra-motor features where both cognitive and behavioural impairments are recognised as part of the condition. Approximately 15% of patients with ALS have concomitant frontotemporal dementia (FTD), but some degree of cognitive or behavioural impairments are present in more than half of patients with ALS, even if criteria for dementia are not fulfilled [1–3]. With support from genetic and neuropathological studies showing common causative mutations and aggregating proteins, ALS and FTD are considered part of the same disease spectrum [4]. Patients with ALS most commonly exhibit a cognitive profile characterised by deficits in executive function, language and social cognition [5–7]. The Edinburgh Cognitive and Behavioural ALS Screen (ECAS) is a well-established screening instrument for cognitive function in ALS and has recently been validated in Swedish [8, 9]. However, previous studies are based on cross-sectional data, and longitudinal reports on ECAS performance over time are lacking [5]. Moreover, patients with ALS and cognitive impairment have shorter survival compared to cognitively intact patients. However, whether cognitive impairment is associated to disease severity and progression has not been investigated [5].

The aim of this study was to investigate the presence and severity of cognitive impairment in ALS at the time of diagnosis and to explore if such impairment was associated with shorter survival, and longitudinal decline in motor symptoms, in a cohort of incident patients with ALS from Sweden. In addition, we aimed to describe the development of cognitive function over time since ALS diagnosis. We hypothesised that cognitive impairment would be present in a significant proportion of patients and associated to prognostic factors. Furthermore, we hypothesised that cognitive function would deteriorate over time in patients with cognitive impairment at the time of diagnosis.

## Materials and methods

### Cohort and data collection

Patients with newly diagnosed ALS were recruited at the ALS Clinical Research Center of the Karolinska University Hospital in Stockholm, Sweden, since 2015. Cognitive data were collected at the time of ALS diagnosis or shortly thereafter (median 5 weeks after diagnosis, henceforward referred to as baseline visit), and longitudinally every 6 months using the validated Swedish version of ECAS and/or Montreal Cognitive Assessment (MoCA) [8–10]. Functional disability was evaluated using the ALS Functional Rating Scale-Revised (ALSFRS-R) [11]. Disease progression rates were defined as the difference in ALSFRS-R scores between two visits (baseline or follow-up), divided by the months between the two time points. The highest overall progression rate was -3 points per month (i.e. this patient lost 18 points between follow-ups) and the median was 0 (i.e., stable cognitive function). In the present study, we included 216 patients diagnosed during 2015–2021 with available measurements on ECAS and/or MoCA. In the majority of patients (*n* = 182), a potential genetic cause of ALS was investigated by screening a panel of genetic variations recognised in neurodegenerative diseases [12].

In total, amongst the 216 patients included in the study, we performed 307 follow-up visits (median number of visits 2, maximum 8). A written informed consent to participate in research was obtained from each participant (or close relative if patient consent were not possible due to severe cognitive impairment). The study was reviewed and approved by the Swedish Ethical Review Authority.

### Cognitive data

ECAS includes cognitive assessments of ALS-specific tasks including language (naming, comprehension, spelling), verbal fluency (fluency letter S and T) and executive function (reverse digit span, alternation, sentence completion, social cognition), and ALS non-specific tasks: memory (immediate recall, delayed recall score, delayed recognition) and visuospatial function (dot counting, cube counting, number location). Composite scores were calculated for each domain (language, verbal fluency, executive function, memory, and visuospatial function). Composite scores were calculated as the sum of the individual test constituting the composite, and the data were scaled to account for the different number of tests included in each composite.

Cognitive impairment was defined as an ECAS total score of 107 or below [8]. When ECAS data were missing (38% at baseline), cognitive impairment was defined as MoCA total score of 25 or below or fulfilling diagnostic criteria for dementia [13].

### Statistical analysis

All data pre-processing, analysis and illustrations were performed in R Studio version 4.3.1. P values below 0.05 were considered statistically significant. Assumptions were assessed visually by residual plots (independence and equal variance) and normal probability plots (normality). Differences in age and ECAS total scores between patients with and without cognitive impairment were assessed by a two-sample *t* test. ALSFRS-R, progression rate and MoCA scores were not normally distributed and differences between groups were, thus, compared using the Wilcoxon rank sum test. Pearson’s Chi-squared test was used to investigate differences in the distributions of sex, site of onset and genetic mutation.

### Baseline cluster analysis

An exploratory cluster analysis was performed on patients with complete data on ECAS composite scores at baseline (*n* = 134) using the K means clustering method [14]. The maximum number of clusters to consider was set to 25. Based on the scree plot (https://en.wikipedia.org/wiki/Scree_plot) and on visual inspection of principal component analysis bi-plots, seven clusters were chosen as the optimal number.

### Survival analysis

Survival after ALS diagnosis was evaluated using Kaplan–Meier plots and Cox proportional hazard models comparing patients with and without cognitive impairment. Survival time was defined as the time from diagnosis to death or invasive ventilation, whichever came first. Patients alive at end of follow-up (September 2021) were censored. The model was adjusted for age, sex, site of onset, mutation status and ALSFRS-R score. These approaches were similarly applied in the exploratory analysis comparing the survival of patients in the different cognitive clusters.

### Longitudinal analysis

A linear mixed-effects model (with random intercept) was applied to examine potential differences in longitudinal trajectories of ECAS and ALSFRS-R scores between patients with and without cognitive impairment at baseline. Due to drop-out in the follow-up, mostly because of disease severity, we chose to limit the analysis from baseline to follow-up at 24 months (in total 216 baseline and 264 follow-up visits, Supplementary Fig. 1). Model selection was based upon clinical relevance and Bayesian information criterion (BIC), where lower BIC was preferred. A stepwise backward selection was performed for the fixed effects using R package lmerTest v3.1–3. Age, sex, months from baseline (0, 6, 12, 18 or 24 months), and cognitive impairment at baseline were included as fixed effects in the final model. In addition, an interaction term between months from baseline (time) and cognitive impairment at baseline was included to investigate whether the trajectories of ECAS and/or ALSFRS-R score were different over time depending on cognitive status at baseline. Patient identifier was included as random effect to account for within-subject correlations.

## Results

### Cognitive impairment at baseline

At the time of ALS diagnosis (baseline), 38% patients had cognitive impairment including 8% that fulfilled criteria for FTD (Table 1). The mean age was higher amongst patients with cognitive impairment compared to those with normal cognitive function (*N* = 216, mean difference 3.4 years, *P* = 2.2e-02). There was a higher proportion of bulbar onset in patients with cognitive impairment than those with normal cognition (*X*^*2*^[2, *N* = 212] = 4.2, *P* = 4.0e-02). Patients with cognitive impairment more often had an causative mutation, mainly driven by individuals with FTD carrying a *C9orf72* repeat expansion within this group (*X*^*2*^[2, *N* = 182] = 9.4, *P* = 2.2e-03). The median ALSFRS-R score was slightly lower in patients with cognitive impairment compared to those with normal cognition (39 and 41, respectively, *P* = 2.4e-02), but the progression rate was similar between the groups. Naturally, since cognitive impairment was defined based on cognitive tests, the median MoCA total score and mean ECAS scores (total, ALS-specific and non-specific) were all different between these two groups.

**Table 1:** Characteristics of the patients included in the study by presence of cognitive impairment at the time of diagnosis

Characteristics	No cognitive impairment (*N* = 133)	Cognitive impairment (*N* = 83)	Total (*N* = 216)	Test statistic	*p* value
Age, mean years (SD)	63.9 (11.4)	67.3 (9.2)	65.2 (10.7)	−2.3	0.02^a^
Sex, F (%)	64 (48.1)	34 (41.0)	98 (45.4)	0.8	0.4^b^
Site of onset, N				4.2	0.04^b^
Bulbar (%)	33 (24.8)	31 (37.3)	64 (29.6)		
Spinal (%)	100 (75.2)	48 (57.9)	148 (68.5)		
FTD (%)	0	4 (4.8)	4 (1.9)		
Genetics, N				9.4	< 0.01^b^
Non carriers (%)	105 (79.0)	49 (59.0)	154 (71.3)		
Mutation carriers (%)	10 (7.5)	18 (21.7)	28 (13.0)		
Unknown (%)	18 (13.5)	16 (19.3)	34 (15.7)		
ALSFRS-R score, median (IQR)	41 (7)	39 (8)	40 (8)	6118.5	0.02^c^
Progression rate, median (IQR)	0.71 (0.7)	0.88 (1.3)	0.76 (0.8)	1015	0.2^c^
ECAS total score, mean (SD)	117.6 (5.3)	97.1 (14.2)	109.0 (14.2)	11.4	< 0.001^a^
ALS-specific, mean (SD)	88.5 (5.0)	74.6 (11.5)	82.8 (10.8)	7.2	< 0.001^a^
MoCA total score, median (IQR)	28 (2)	25 (4)	27 (3)	8537	< 0.001^c^

^a^ Two-sample *t* test

^b^ Pearson’s Chi-square test. Site of onset: bulbar vs. spinal onset (FTD was excluded). Genetics: non-carriers vs. mutation carriers (unknown was excluded)

^c^ Wilcoxon rank sum test

The cluster analysis identified three main cognitive clusters at baseline (Supplementary Figs. 2 and 3). Cluster 1 (23%) included patients with impairment primarily in executive function and language. Cluster 2 (10%) included patients with impairment in verbal fluency whereas patients in Cluster 3 (10%) presented a more general cognitive impairment but with spared verbal fluency. The remaining clusters comprised patients with normal cognitive function (reference, 51%), patients with impairment in social cognition (Cluster 4), and two clusters including only three patients (Clusters 5 and 6). There was no difference regarding patient characteristics between the clusters, including age, sex, site of onset, genetic cause and ALSFRS-R score at baseline. However, patients in Cluster 3 had the shortest survival (median survival time 41.2 weeks compared to 65.7 and 72.4 weeks in Clusters 2 and 1, respectively) (Supplementary Fig. 4).

### Cognitive function and survival

**Fig. 1:**
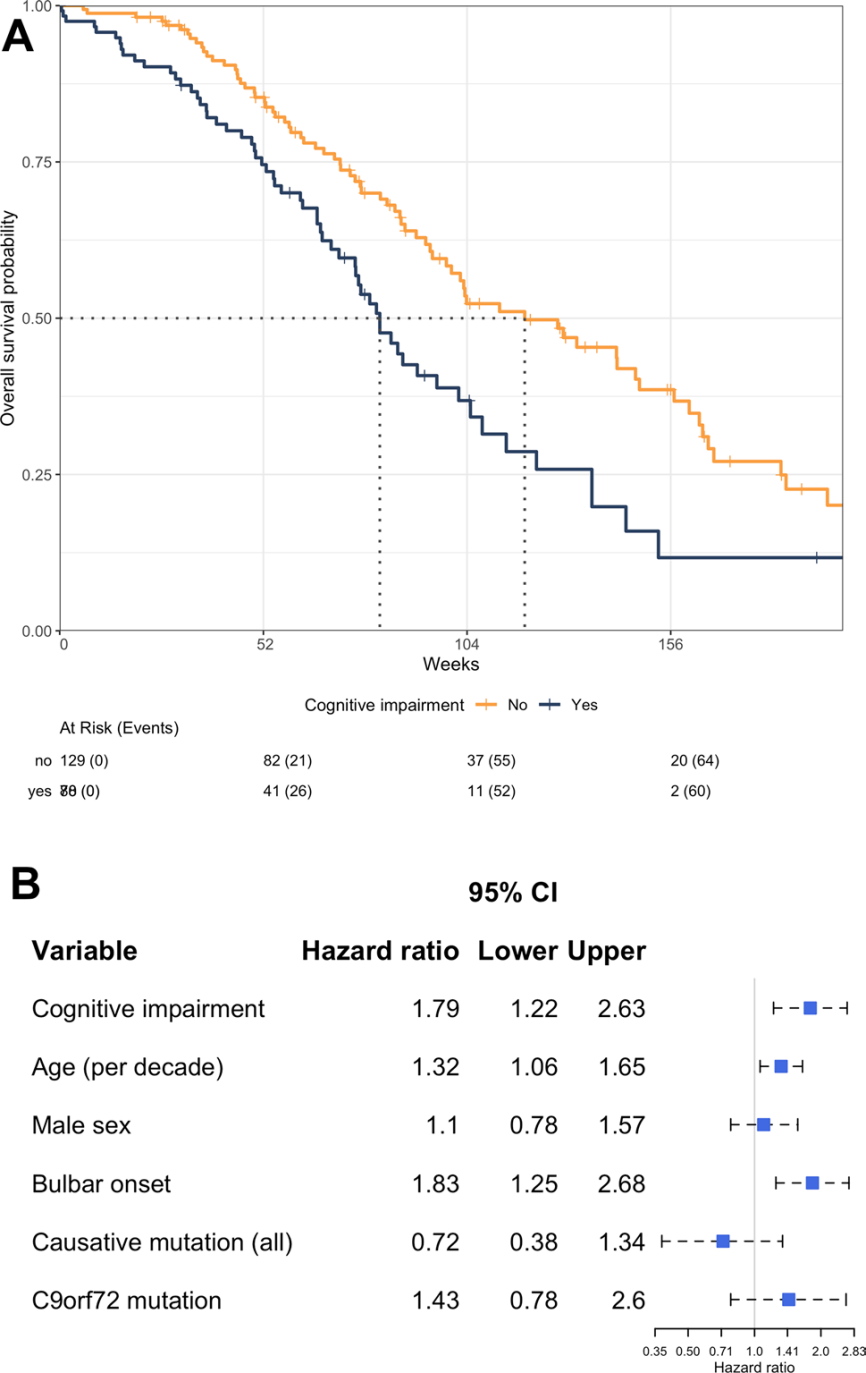
Cognitive impairment and ALS survival. Dotted lines = median survival. CI = confidence interval. *C9orf72* = chromosome open reading frame 72. **A** Kaplan–Meier plot showing ALS survival by cognitive status. **B** Hazard ratios and 95% confidence intervals of death in relation to patient characteristics derived from Cox proportional hazard models

Cognitive impairment at baseline was associated with a shorter survival (Fig. 1A) and a higher risk of death during follow-up, after multivariable adjustment (hazard ratio 1.79, 95% CI 1.22–2.63, *P* = 2.9e-03) (Fig. 1B). The median survival time in patients with cognitive impairment was 65.7 weeks, compared to 94.6 weeks in patients without cognitive impairment (dotted lines in Fig. 1A). A higher age, but not sex, was associated with shorter survival (hazard ratio 1.32, 95% CI 1.06–1.65; per 10 years, *P* = 1.3e-02 and hazard ratio 1.1, 95% CI 0.78–1.57, comparing female to male, *P* = 5.8e-01, respectively). In addition, patients with bulbar onset had shorter survival (hazard ratio 1.83, 95% CI 1.25–2.68, *P* = 1.9e-03). The presence of a causative mutation was not associated with risk of death.

### Longitudinal cognitive function

**Fig. 2:**
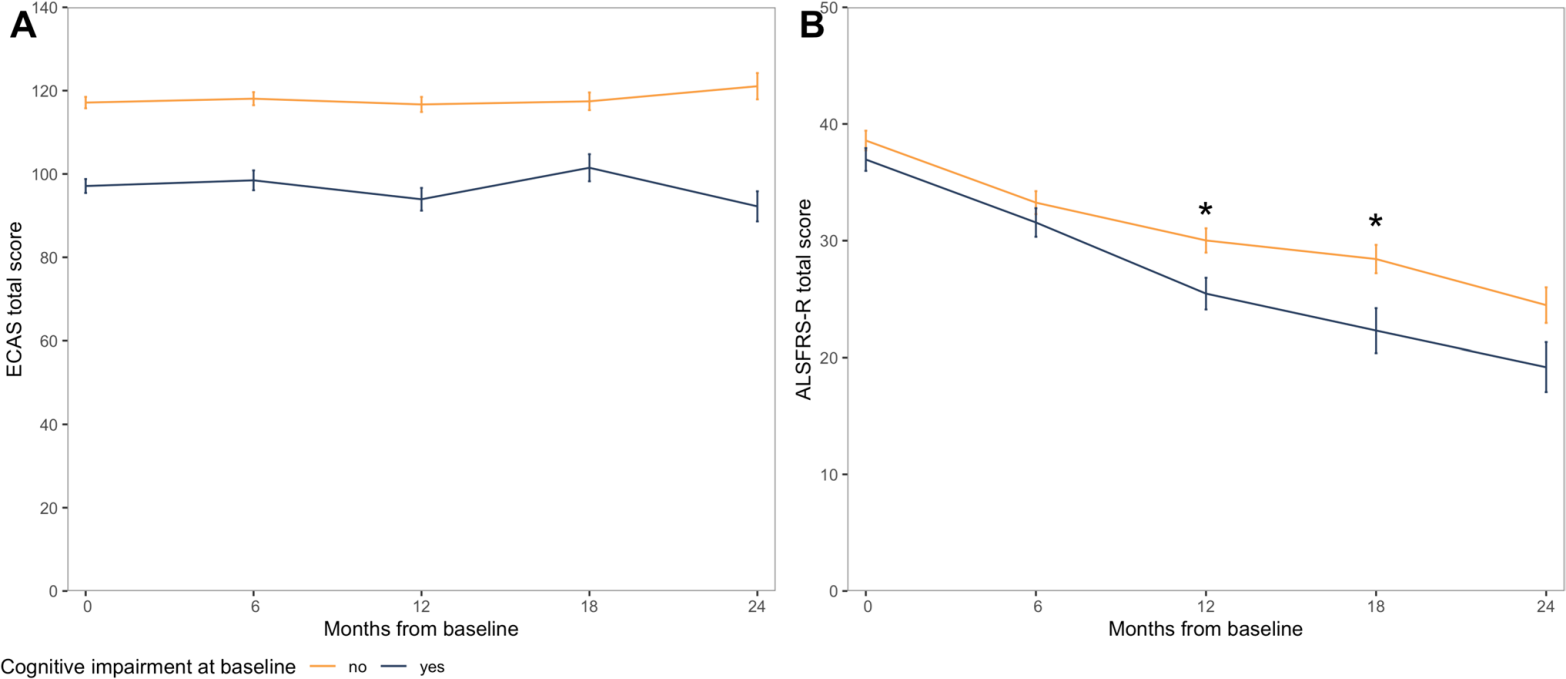
Cognitive and motor functions over time in patients with and without cognitive impairment at the time of diagnosis. Linear mixed effect models adjusted for age and sex. Point = mean value at each time point. Error bars = mean minus standard error. Asterisks = statistically significant different trajectories of ALSFRS-R between patients with and without cognitive impairment. **A** ECAS total scores. **B** ALSFRS-R scores

Overall, ECAS scores did not decrease considerably during the first 2 years of follow-up (Fig. 2A), in clear contrast to the persistently declining ALSFRS-R scores (Fig. 2B). The majority (80%) of patients with normal cognition at baseline remained cognitively intact along the disease course. Having cognitive impairment at baseline was associated with a more rapid decline in ALSFRS-R score at 12- and 18-month follow-up (Fig. 2B). Estimates and *P* values for the statistical tests are shown in Supplementary Table 1.

To investigate potential bias in the longitudinal analysis due to informative dropouts, we conducted a sensitivity analysis including patients with four or more visits (i.e. follow-up for at least 18 months, *N* = 44). The trajectories of both ECAS and ALSFRS-R scores were similar to the ones seen in the main analysis (Supplementary Fig. 5A and B). In addition, there were no statistically significant differences in follow-up rates between patients with and without cognitive impairment at baseline, indicating comparable rates of continued participation regardless of cognitive status.

Similar patterns of stable cognitive performance over time were seen when investigating ALS-specific, non-specific and domain-specific ECAS scores (Supplementary Figs. 6 and 7).

## Discussion

To evaluate cognitive function over time in patients with ALS, we performed ECAS assessments in a large population of newly diagnosed patients with ALS from Sweden, including follow-up evaluations every 6 months. We found that 38% of the patients had cognitive impairment early on in the disease. Similar to previous results, the majority of these patients had deficits in executive function and/or the different language domains, which are the domains constituting the ALS-specific scores in ECAS [15, 16].

Interestingly, cognitive function was stable over time, and did not follow the trajectories of declining motor functions by time since ALS diagnosis. Earlier studies have demonstrated that patients with ALS and normal cognition at disease onset are unlikely to develop cognitive impairment during the disease [17, 18]. However, here we show that the cognitive function is stable during the first 2 years after diagnosis also in patients with cognitive impairment at the time of diagnosis, conversely to what we first anticipated. Whether cognitive function would continue to be stable beyond 2 years after diagnosis (primarily amongst patients with slow progressing ALS), or if cognitive function would deteriorate albeit at a slower rate than motor functions and thus not captured during our follow-up, remains to be established. Some studies support the former, arguing that cognitive impairment in ALS is a distinct phenotype, and not necessarily a consequence of disease progression [19]. However, longitudinal studies on deterioration of specific cognitive domains have shown contradictory results [19]. Some reports suggest a decline in executive function and language over time in patients with ALS [18, 20], whilst others found no progressive deterioration in these domains [21–23]. It should be noted that our results are at group level and individual variability was observed. In this cohort, demographic and clinical characteristics (age, sex distribution, disease severity, site of onset and presence of a genetic mutation) were similar between patients with an ECAS progression rate above vs below 0.25 points/months (data not shown). Going forward, other factors potentially contributing to an increased susceptibility to develop cognitive impairment in ALS should be explored as well as associations to anxiety, depression, behavioural symptoms and fluid biomarker levels amongst other things.

In our study, both ALS-specific (including executive function and language) and non-specific functions remained stable over time. The domain-specific scores did not show significant temporal trends during the follow-up either. Consequently, we did not observe a statistically important practice effect amongst patients with ALS regardless of cognitive status. Practice effect is the improvement in neuropsychological test scores typically found when a person is administered the same tests repeatedly. A lack of practice effect has previously been found in several neurodegenerative diseases including ALS [24, 25]. In addition, loss of practice effect is implicated as a potential biomarker in preclinical FTD due to *C9orf72* repeat expansions [26]. Whether the stable ECAS performance over time presented in our study is due to the absence of a practice effect, or an actual cognitive deterioration masked by practice effect, is not possible to distinguish with the current study design.

Even if cognitive function is not declining as ALS progresses, our results imply that cognitive impairment at the time of diagnosis is associated with a more rapid decline of motor functions. As expected, ALSFRS-R deteriorated over time in all patients. However, the gradient was steeper in those with cognitive impairment at baseline. In addition, cognitive impairment was associated with a shorter survival in ALS. Patients with normal cognition had a median survival of around 8 months longer than patients with cognitive impairment. Patients with a general cognitive impairment profile but with spared verbal fluency (Cluster 3) appeared to have the shortest survival.

The strength of this study is the large patient material including a complete and long follow-up. To our knowledge, this is the first study investigating repeated ECAS assessments. On the other hand, this could be considered a limitation as the sensitivity to detect longitudinal cognitive change might be limited which would explain the lack of deterioration in our cohort. We also acknowledge other limitations. In longitudinal studies of ALS, there is a risk of selection bias as patients with better prognosis are more likely to contribute repeated assessments. In an attempt to explore the potential impact of such bias, we performed a sensitivity analysis including only patients with at least four ECAS assessments, showing the same results as in the main analysis. Also, drop-out rates were similar between patients with and without cognitive impairment. However, we do not know whether the non-participating patients would be more likely to have cognitive impairment. Finally, both ECAS and MoCA are screening tools and may have limited ability to detect subtle cognitive impairment, especially in certain domains [9, 27]. On the other hand, extensive neuropsychological assessments in very vulnerable populations such as patients with ALS require particular consideration, and less burdensome tools to evaluate cognitive function other than conventional testing are needed. Investigating the potential of repeated ECAS is, therefore, important. Due to the limitations of ECAS when studying specific cognitive domains, the cluster analysis is exploratory and should be interpreted as such. In addition, K means clustering is sensitive to outliers, and the algorithm prefers clusters of more or less equal sizes. To evaluate the stability of the clustering results, we repeated the algorithm ten times with different seeds, generating similar results each time (data not shown).

In summary, cognitive impairment is a frequent and early event in ALS, with a suggested negative impact on disease progression and survival. Cognitive function seems to remain stable over time for both patients with and without cognitive impairment at diagnosis. However, we do not know the mechanisms underlying why certain patients develop cognitive impairment whilst others have spared cognitive function. Cognitive and behavioural symptoms, rather than physical disabilities, are reported by caregivers as the most difficult to handle in ALS [28]. We, therefore, advocate for a systematic monitoring of cognitive function to be able to provide proper information of cognitive symptoms, prognostication, estimated functional decline and adequate personalised support to patients and their families.

## Supplementary Information

Below is the link to the electronic supplementary material.

Supplementary file1 (DOCX 26398 KB)

## Declarations

### Availability of data and materials

The dataset is available from the corresponding author upon reasonable request.
