# Correction: Repeated cognitive assessments show stable function over time in patients with ALS

**DOI:** 10.1007/s00415-024-12833-z

**Published:** 2025-01-08

**Authors:** Linn Öijerstedt, Juliette Foucher, Anikó Lovik, Solmaz Yazdani, Alexander Juto, Ulf Kläppe, Fang Fang, Caroline Ingre

**Affiliations:** 1 Department of Clinical Neuroscience, Karolinska Insitutet, 171 77 Stockholm, Sweden; 2 Department of Neurology, Karolinska University Hospital, Stockholm, Sweden; 3 Institute of Environmental Medicine, Karolinska Institutet, Stockholm, Sweden; 4 Institute of Psychology, Leiden University, Leiden, The Netherlands

**Correction: Journal of Neurology (2024) 271:5267–5274** 10.1007/s00415-024-12479-x

In the original version of this article, the wrong Supplementary file was originally published and it has now been replaced with the correct file.

The original article has been corrected.

## Supplementary Information

Below is the link to the electronic supplementary material.

Supplementary file1 (DOCX 26397 kb)

