# Supplementary material for: Repeated cognitive assessments show stable function over time in patients with ALS
Source: J Neurol. 2024 Jun 9;271(8):5267–74. doi: 10.1007/s00415-024-12479-x (PMC11319384; doi:10.1007/s00415-024-12479-x)

**SUPPLEMENTARY MATERIAS**

**Repeated cognitive assessments show stable function over time in patients with ALS**

Linn Öijerstedt^a,b*^, Juliette Foucher^a,b^, Anikó Lovik^c,d^, Solmaz Yazdani^a^, Alexander Juto^a,b^, Ulf Kläppe^a,b^, Fang Fang^c^, Caroline Ingre^a,b^

^a^ Department of Clinical Neuroscience, Karolinska Insitutet, Stockholm, Sweden

^b^ Department of Neurology, Karolinska University Hospital, Stockholm, Sweden

^c^ Institute of Environmental Medicine, Karolinska Institutet, Stockholm, Sweden

^d^ Institute of Psychology, Leiden University, Leiden, The Netherlands

**Supplementary Figure 1.** Number of participants at each time point.


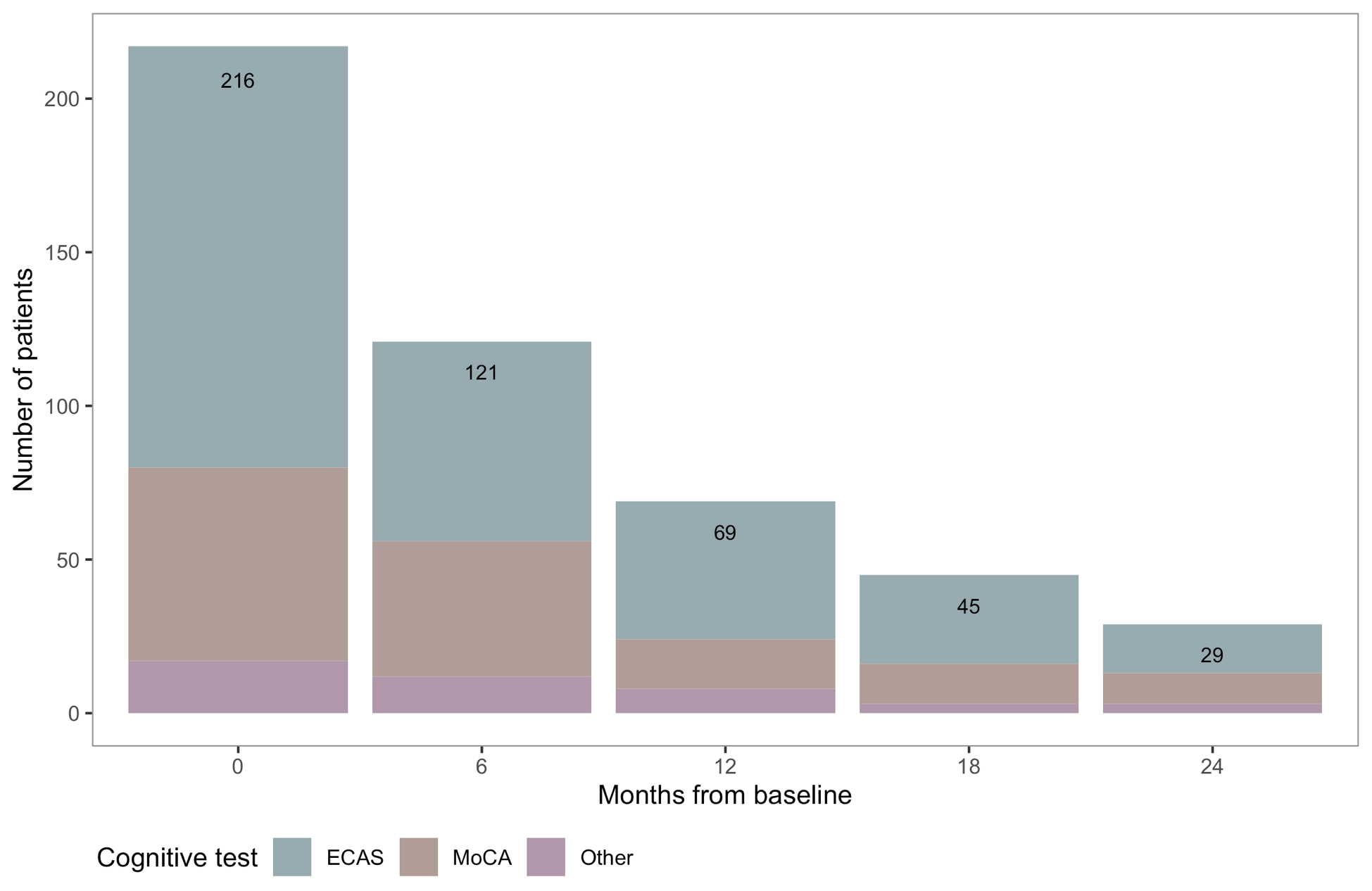


Other = Fulfilling criteria for dementia

**Supplementary Figure 2.** Characteristics of cluster 1-3:


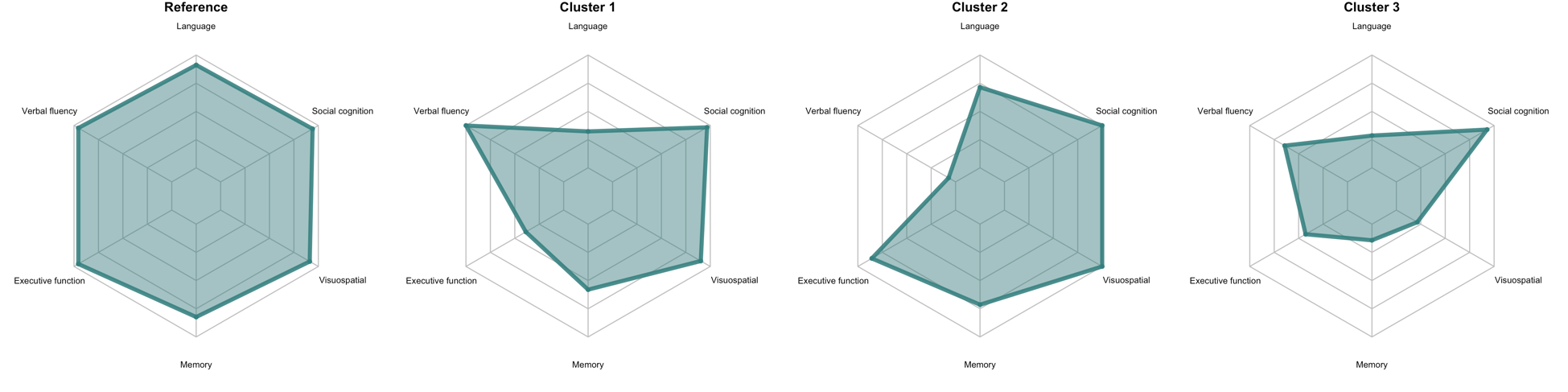


**Supplementary Figure 3.** Distribution of cognitive status among clusters:


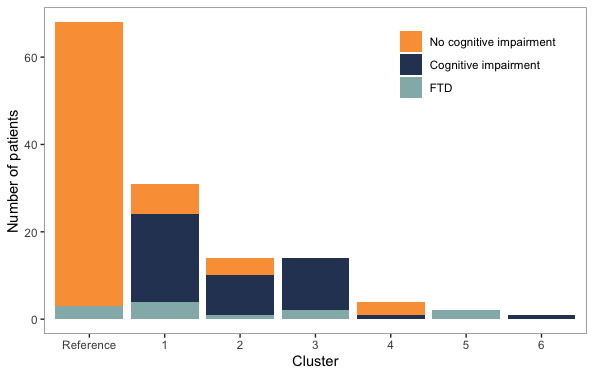


**Supplementary Figure 4.** Hazard ratios and survival based on clusters:


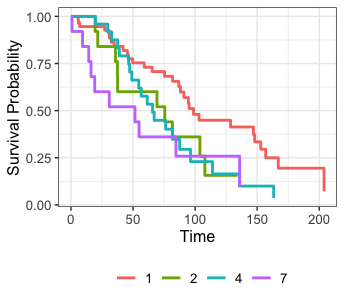
A. B.


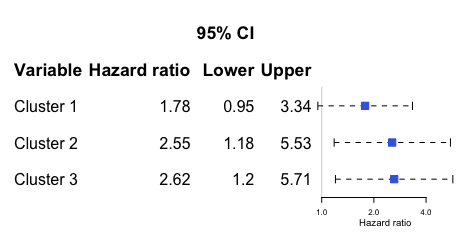


Red = reference/normal, blue = cluster 1, green = cluster 2, purple = cluster 3

**Supplementary Table 1**. Output from linear mixed effect model. BL=baseline.

|  | **ALSFRS-R total score** | | | | | | **ECAS total score** | | | | |
| --- | --- | --- | --- | --- | --- | --- | --- | --- | --- | --- | --- |
| **Fixed effects** | **Estimate** | **SE** | **df** | **t value** | **p** | **Estimate** | | **SE** | **df** | **t value** | **Pr(>\|t\|)** |
| (Intercept) | 45.6 | 2.8 | 217.5 | 16.1 | 7.8e-39 | 122.0 | | 4.4 | 118.3 | 27.9 | 7.3e-54 |
| Age | -0.1 | 0.0 | 213.6 | -2.3 | 2.0e-02 | -0.1 | | 0.1 | 114.7 | -1.2 | 0.2 |
| Male sex | 0.5 | 0.9 | 216.8 | 0.5 | 0.6 | -0.7 | | 1.5 | 128.5 | -0.5 | 0.6 |
| Cognitive impairment at BL | -1.6 | 1.0 | 305.3 | -1.6 | 0.1 | 0.9 | | 1.4 | 151.9 | 0.7 | 7.2e-24 |
| Time 6 months from BL | -5.3 | 0.8 | 272.8 | -6.9 | 4.0e-11 | -0.4 | | 1.7 | 156.6 | -0.3 | 0.5 |
| Time 12 months from BL | -8.6 | 0.8 | 273.4 | -10.1 | 1.5e-20 | 0.3 | | 2.0 | 147.3 | 0.2 | 0.8 |
| Time 18 months from BL | -10.2 | 1.0 | 263.9 | -9.7 | 3.3e-19 | 3.9 | | 3.1 | 201.7 | 1.3 | 0.9 |
| Time 24 months from BL | -14.1 | 1.4 | 258.2 | -10.1 | 1.6e-20 | -20.0 | | 1.8 | 217.9 | -11.4 | 0.2 |
| Interaction between Time and cognitive impairment at BL at: |  |  |  |  |  |  | |  |  |  |  |
| 6 months | -0.1 | 1.3 | 276.6 | -0.1 | 1.0 | 0.4 | | 2.6 | 170.7 | 0.2 | 0.9 |
| 12 months | -2.9 | 1.5 | 272.4 | -2.0 | 4.9e-02 | -2.7 | | 3.1 | 154.9 | -0.9 | 0.4 |
| 18 months | -4.5 | 2.1 | 263.6 | -2.1 | 3.3e-02 | 4.1 | | 3.8 | 152.8 | 1.1 | 0.3 |
| 24 months | -3.7 | 2.5 | 260.9 | -1.5 | 1.4e-01 | -8.8 | | 4.8 | 204.8 | -1.8 | 0.1 |

Reference is the score at baseline for a female with no cognitive impairment.

**Supplementary Figure 5.** Sensitivity lme analysis (patients with four or more visits, i.e. follow-up for at least 18 months, N=44):


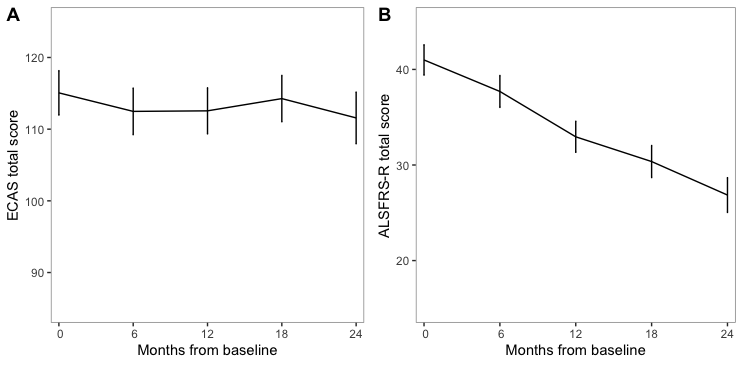


**Supplementary Figure 6.** ECAS ALS specific and non-specific scores over time (scaled and adjusted for age and sex).


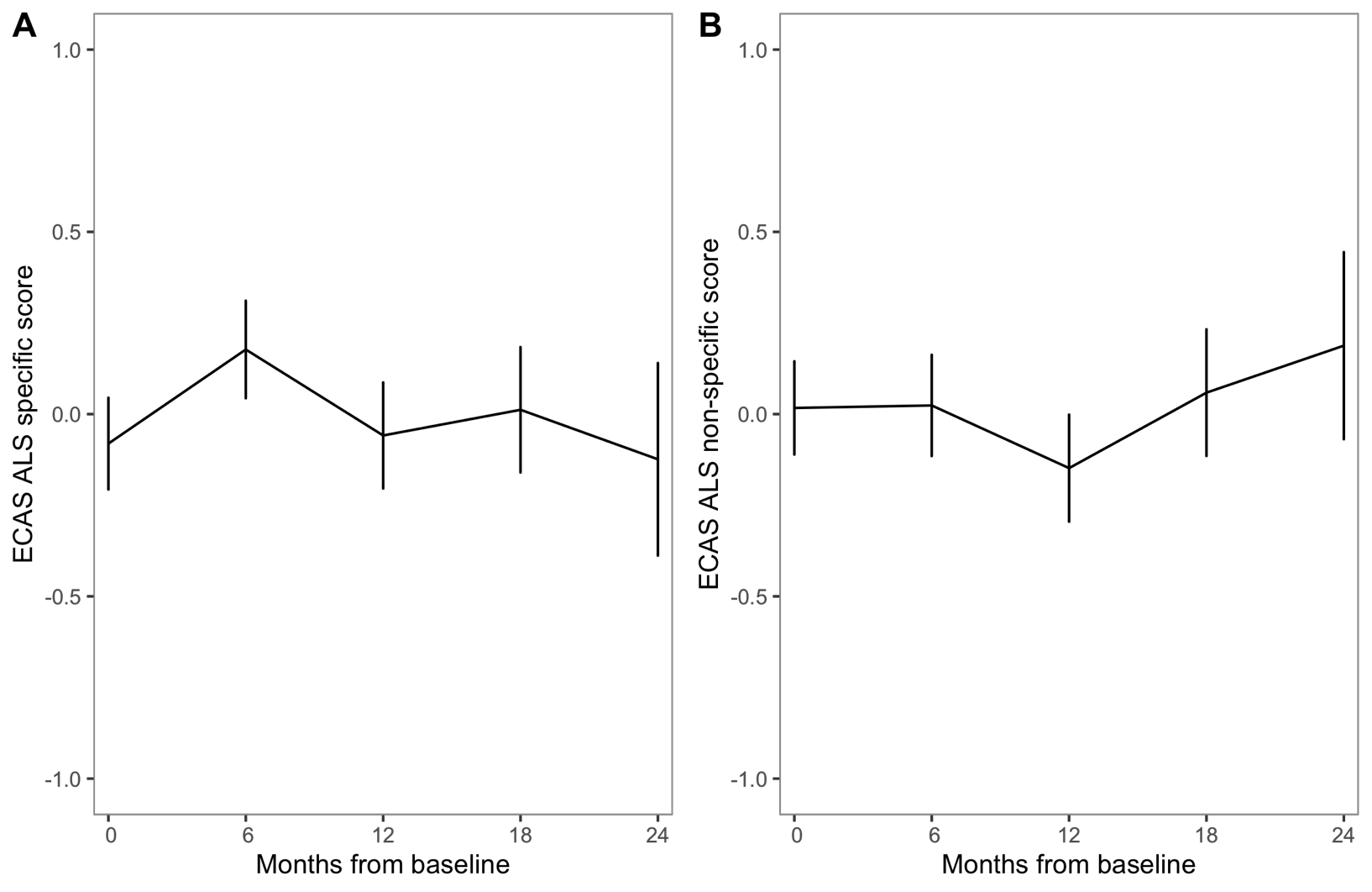


**Supplementary Figure 7.** ECAS sub domain scores over time (scaled and adjusted for age and sex).


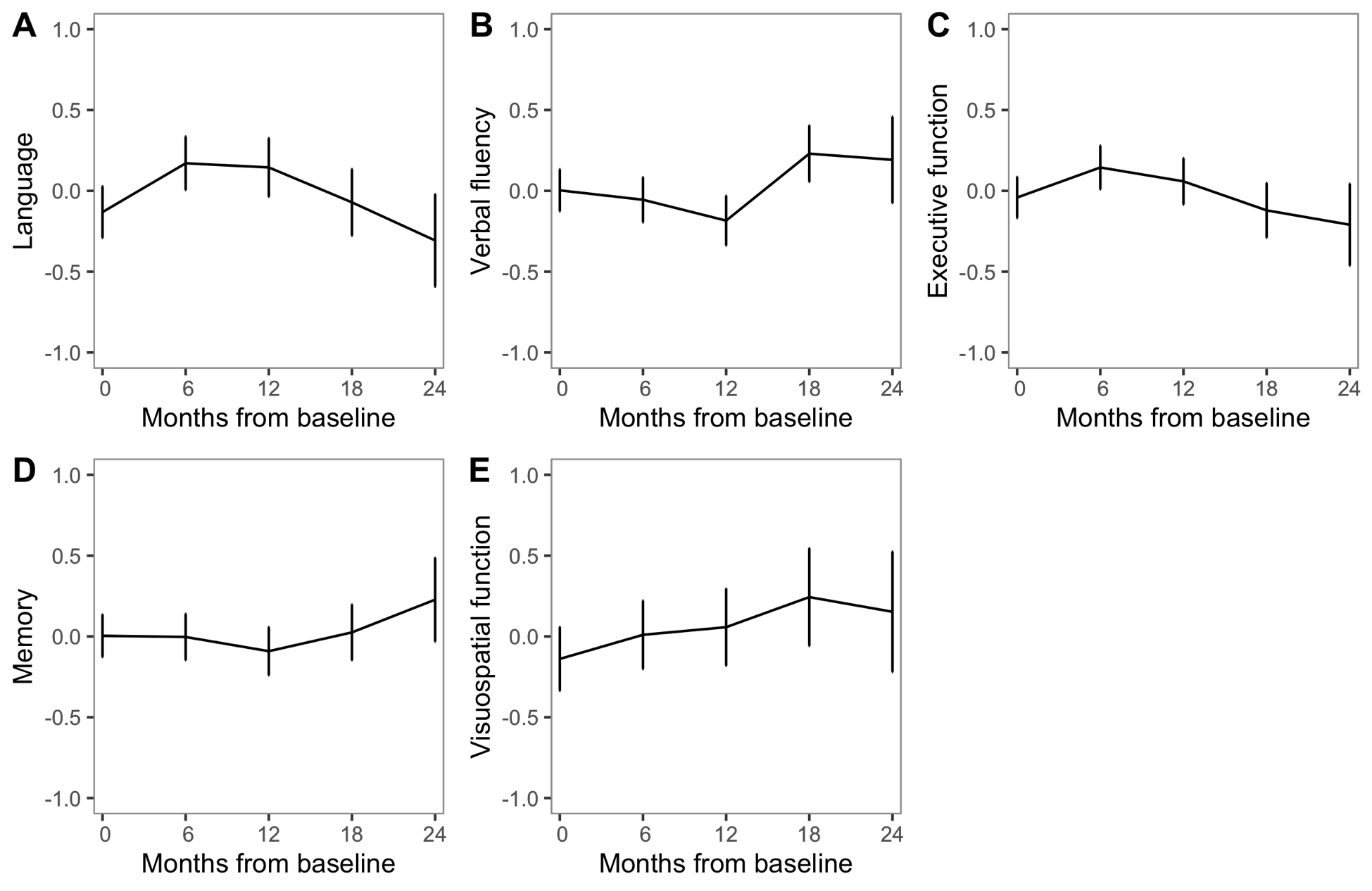

Supplement: Supplementary file 1 — Supplementary file1 (DOCX 26398 KB) [file 415_2024_12479_MOESM1_ESM.docx]
